# What the World Is Doing

**Published:** 1895-08

**Authors:** 


### WHAT THE WORLD IS DOING.

The Argentine Republic is now shipping beef to England, and it is claimed that they can undersell American beef, though the meat is inferior in quality to that American grown.

An outbreak of anthrax is reported at Newport, near Bridgeton, N. J., by Veterinarians Rogers and Hewitt.

England's Diseases of Animals Act of 1894 is so constituted that a police-officer may be clothed with power to decide the question of quarantine, and to declare infected premises free from contagion or infection. Surely this is a grievous blunder, and an insult to the intelligence of the veterinary profession of Great Britain.

State Veterinarian Trumbower reports an outbreak of Texas fever in Southern Illinois.

One of South New Jersey's formerly leading veterinarians has removed to Philadelphia, where he has opened a barber-shop in connection with the practice of the calling.

The Iowa State Veterinary Medical Association steps forward among the first with a proposition that after January 1, 1895, all applicants for membership shall be graduates of colleges having a curriculum of three years of not less than six months each. This movement will surely be followed soon by others. Will not the veterinary schools maintaining the short-term sessions bend rather than be broken?

Harper's *Monthly* for July, in a well-prepared article descriptive of the University of Pennsylvania, among other handsome illustrations, has two of the Veterinary Department. The picture of the Canine Hospital is very good and quite suggestive of the purposes of this department.

St. Louis, Missouri, will inaugurate a good movement in issuing licenses under good rules to all dairies sending milk into that city.

The State Board of Health of California advises all local boards to take active measures to prevent the sale from tuberculous herds.

A dairy upon strictly sanitary principles has been established at Independence, Missouri, for the production of a pure-milk supply. Sterilized milk and prepared milk-combinations on physician's prescriptions will be a feature of the undertaking. Veterinarian Wattles, of Kansas City, has been retained as examining surgeon of the cattle and director of the dairy.

Veterinarians will be glad to learn that Messrs. Teufel & Co., instrument-makers of Philadelphia, have brought out a satisfactorily-working veterinary thermo-cautery. To some who have had annoying experiences with those placed on the market the past two or three years this will be grateful news.

Loud and just are the complaints of veterinarians of London, England, at the lack of recognition conceded them by the governing authorities. Agitation is the watchword of the hour the world over for the veterinarian. His position in the sanitary field of science stands second to none.

The Iowa State Veterinary Medical Association has placed the entire proceedings of the meetings of 1894 in book form, together with the papers, discussions, etc. Among her committees for 1895 we note with pleasure those of army legislation and the U. S. V. M. A. meeting in their State.

At the annual Sanitary Convention of California the local Board of Health of San Francisco was advised to consult with the veterinary faculty of the California College as to the best methods of inspection of dairies and dairy products. Quite right, gentlemen, this is the province of the veterinarian, and his education well fits him for this special line of work.

Colorado will exact a thorough inspection of all Mexican cattle entering the borders of that State from now until December 1, 1895.

B. A. Walton, of Atlanta, Georgia, died recently of hydrophobia, though he had undergone treatment at the Pasteur Institute at New York City, and was discharged as cured.

Anthrax, the fatal disease among cattle at Newport, N. J., has reached the vicinity of Cedarville, and many farmers have lost cows and horses.

The Board of Regents of New York State have appointed the following members of the profession State Examiners in compliance with the law passed at the recent session of the State Legislature: Professors James Law, R. S. Huidekoper, Drs. Nelson P. Hinkley, William H. Kelly, and Claude D. Morris. This board will convene for the first time on September 24th and 27th next to make the necessary plans for inauguration of the provisions of the act.

The bill which passed both houses of the Legislature in New Hampshire, appropriating $100,000 for the suppression of tuberculosis, has been vetoed by the Governor.

